# Thirty years on: HIV receptor gymnastics and the prevention of infection

**DOI:** 10.1186/1741-7007-11-57

**Published:** 2013-05-21

**Authors:** Robin A Weiss

**Affiliations:** 1 Wohl Virion Centre, Division of Infection & Immunity, University College London, Gower Street, London WC1E, UK

**Keywords:** AIDS, HIV, cell receptors, CD4, CCR5, CXCR4, Therapy

## Abstract

During 30 years of research on human immunodeficiency virus (HIV), our knowledge of its cellular receptors - CD4, CCR5 and CXCR4 - has illuminated aspects of the pathogenesis of the acquired immune deficiency syndrome (AIDS). Studying how the HIV envelope glycoproteins interact with the receptors led to anti-retroviral drugs based on blocking the docking or fusion of virus to the host cell. Genetic polymorphisms of CCR5 determine resistance to HIV infection and the rate of progression to AIDS. Eliciting neutralizing antibodies to the sites of receptor interaction on HIV glycoproteins is a promising approach to HIV vaccine development.

## Opinion

As we celebrate the 30th anniversary of the discovery of HIV-1 by Françoise Barré-Sinoussi and colleagues [1], it is a sobering thought that during the two years between the first notification of AIDS [2] and the discovery of its cause, epidemiologists established all the modes of transmission and risk factors associated with HIV-1 infection without knowing the identity of the virus. Moreover, it became apparent from the first full clinical description of AIDS in 1981 [3] that the salient feature underlying the disease is a specific depletion of CD4 T-helper lymphocytes. So was the 1983 discovery of HIV-1 in Paris and its confirmation a year later by new isolations from AIDS patients in Africa [4], and America [5,6] such a great leap forward? Most certainly yes!

First, the identification of HIV-1 rapidly led to diagnostic tests of infection that made blood donations safe again. Second, the recognition of HIV as a retrovirus led to the development of anti-retroviral drugs that have so dramatically reduced AIDS mortality and morbidity where treatment is available. Third, the measurement of virus load (the number of HIV genomes in the plasma) became an important prognostic marker, alongside CD4 T-cell counts, for monitoring the health of patients. Fourth, analyses of HIV informed us early on of ineffective immune responses to it [7,8] and of the enormous antigenic diversity of the virus [9], which have been stumbling blocks in the development of a broadly efficacious vaccine.

## HIV envelope glycoproteins

**Figure 1:**
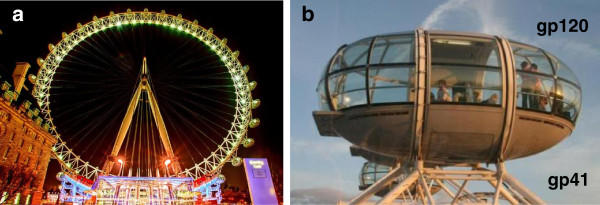
**The London Eye as a ’model’ of HIV.** (**a**) The model depicts the diploid RNA genome and the outer envelope of the HIV particle composed of a lipid bilayer studded with glycoprotein spikes. (**b**) Close-up of trimeric spike showing globular gp120 and transmembrane gp41.

The 135 m diameter ferris wheel called the London Eye (Figure 1a) is an apt model of a cross-section of the HIV particle magnified by 10^9^. The viral envelope is studded with viral glycoprotein ‘spikes’ that recognize the cell receptors to which HIV binds as the first step in virus entry. Each spike (Figure 1b) has a trimeric structure comprising three transmembrane glycoproteins (gp41) coupled to three surface glycoproteins (gp120) named after their approximate molecular weights of 41,000 and 120,000 Da, respectively. Gp120 is heavily glycosylated, particularly with N-linked high-mannose residues and the carbohydrates make up more than 50% of its mass [10]. They form a glycan shield or carapace protecting sensitive sites such as receptor-binding pockets, but also present epitopes recognized by some neutralizing antibodies [11].

Figure 1 is deceptive, however, in that the envelope spikes are actually flexible structures that undergo important conformational rearrangement during binding and entry into cells [12]. In order to enter cells, HIV must bind to CD4, the signature cell-surface marker of T-helper cells, and to one of the chemokine receptors characteristic of these cells. Gp120 bears the binding sites for the CD4 viral receptor and chemokine co-receptors, while gp41 contains the hydrophobic domains that effect fusion between viral envelope and host membranes.

## The CD4 receptor and the shape of viral recognition

The discovery that CD4 is the HIV receptor followed soon after the description of HIV-1. As already mentioned, destruction of CD4-positive T-helper lymphocytes was highlighted in an early report [3] of the nature of the immunodeficiency, which was subsequently called AIDS. This selective depletion of CD4+ cells was replicated *in vitro* by David Klatzmann and colleagues, who sorted CD3+ T cells into CD4+ and CD8+ enriched fractions and observed that HIV replicated in and destroyed only the CD4+ population [13]. These findings did not necessarily imply that the very same cell surface maker that immunologists use to type T-cell subsets would be recognized by HIV. However, that proved to be the case when monoclonal antibodies specific to CD4 were found to competitively block HIV infection *in vitro*[14,15]. The binding epitope on CD4 was later mapped via monoclonal antibodies, site-specific mutagenesis and structural studies to be located on the amino-terminal domain, for which a phenylalanine residue at position 43 is crucial for binding in a gp120 pocket [16].

CD4 is a single chain class I membrane glycoprotein of the immunoglobulin superfamily. Five years after the recognition of CD4 as the HIV receptor, two other members of this family were identified as viral receptors, namely the adhesion molecule ICAM-1 for the major group of rhinoviruses [17] and a related molecule for all three serotypes of poliovirus [18]. The binding sites for these receptors are buried in deep pockets or clefts in the viral proteins that bind them, as is the sialic acid binding site on the hemagglutinin of influenza viruses. This led Michael Rossman to postulate the canyon hypothesis [17] that viruses adopt this kind of receptor because bulky antibodies bearing both heavy and light chains at the antigen-binding sites cannot penetrate into the canyon on the virus. This consideration led us to exploit llama heavy-chain only antibody fragments, which should be able to gain access to the canyon in a similar way to CD4 itself. However, some potently neutralizing conventional human antibodies like VRC01 also recognize the CD4 binding site so penetration deep into the pocket may not be essential [11].

## Chemokine coreceptors and resistance to HIV

As well as infecting T-helper lymphocytes, HIV can infect macrophages and other cells of the same lineage, such as microglia in the brain, which express low levels of CD4. It soon became clear, however, that CD4 was necessary but not sufficient for HIV infection, as expression of human CD4 on murine cells did not confer susceptibility to HIV entry [19]. It took a further ten years before seven-transmembrane chemokine receptors were identified as the missing component or co-receptor, with a landmark paper [20] on CXCR4 serving as the co-receptor for cell-line adapted strains of HIV-1. Three months before the discovery of CXCR4 as a co-receptor for HIV, it had been reported that CCL3L1 (MIP-1αP), CCL4 (MIP-1β) and CCL5 (RANTES) could block infection [21]. The only receptor that binds all three of these chemokines is CCR5. With this clue and the discovery of CXCR4, several groups quickly demonstrated that CCR5 is the co-receptor for HIV strains that infect primary T cells and macrophages [12].

CCR5-using (R5) viruses represent the major transmissible HIV-1 strains, whereas CXCR4-using (X4) viruses tend to arise late in the course of disease. X4 viruses are often thought to precipitate AIDS, but they occur only in a minority of AIDS patients. Turning the argument on its head, we suggested that while X4 viruses are ill-adapted for propagation in healthy individuals, they emerge as opportunistic HIV variants once immunodeficiency begins to set in [22].

The discovery of CCR5 as a co-receptor rapidly led to the identification of a major genetic resistance factor for HIV infection. It had been a puzzle that some highly exposed sexual partners of HIV-infected individuals had managed to escape infection. It was shown that one such patient was a natural CCR5 knock-out, being homozygous for a deletion of 32 base pairs in exon 1 of the CCR5 gene (Δ32), and his cells could not be infected *in vitro*[23]. The Δ32 allele occurs relatively frequently in Caucasian populations and while heterozygotes are not wholly resistant to HIV, they are less susceptible to infection, and once infected, they progress to AIDS at a slower rate than people with wild-type CCR5. An HIV-infected individual with leukemia who received a bone marrow stem cell transplant from a CCR5-negative donor appears to have slowly eliminated his virus [24]. Other CCR5 polymorphisms in the promoter region also occur in non-Caucasian populations and affect susceptibility to HIV and progression to AIDS [25]. The density of expression of CCR5 on lymphocytes and the plasma concentration of chemokines that bind to it act in concert to modulate efficiency of HIV entry.

A number of other chemokine receptors can act as functional co-receptors for HIV *in vitro* but there is scant evidence that they play a role *in vivo*[12]. Of more practical significance is the discovery that HIV binds to the adhesion molecule DC-SIGN on dendritic cells [26]. DC-SIGN does not serve as a receptor for virus entry, but dendritic cells migrating from mucosal tissues to lymph nodes and bearing HIV particles on the surface provide a route whereby the virus can be delivered to susceptible CD4+ CCR5+ T cells in the lymph nodes.

## The gymnastics of fusion and entry of virus into cells

**Figure 2:**
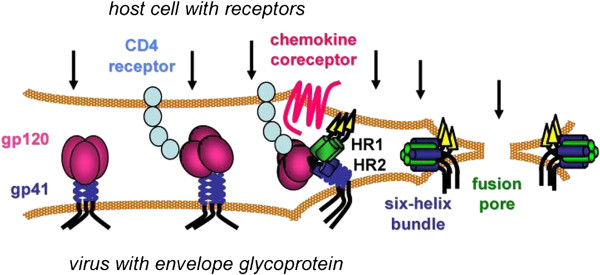
**Model of HIV entry.** CD4 receptors and chemokine co-receptors are shown on the host cell. The gp120 surface subunit and gp41 transmembrane subunit of the HIV envelope glycoprotein are shown on the viral membrane (envelope). After gp120 binds to CD4, the envelope glycoprotein undergoes conformational changes that facilitate gp120 interaction with the chemokine co-receptor. Additional conformational changes in the gp41 transmembrane subunit transiently expose two heptad-repeat domains (HR1 and HR2) that subsequently self-assemble to form a six-helix bundle structure. Formation of several gp41 six-helix bundles bring the host and viral membranes together for fusion, while several six-helix bundles likely coalesce to form a fusion pore that allows the viral core to pass into the host cell cytoplasm. Arrows indicate potential steps in the entry process for inhibition. (Reproduced from [32] with kind permission of the authors).

HIV entry involves a stepwise series of interactions with receptors that initiate conformational changes in the envelope glycoproteins [12,27] (Figure 2). Docking on to CD4 induces a conformational change in gp120 that exposes a site known as the CD4 induced site (CD4i) and allows the protrusion of one of three hypervariable loops of gp120 (variable loop 3, or V3 loop). Both CD4i and the V3 loop interact with chemokine receptors, the V3 loop being the major determinant of R5 and X4 tropism. At the same time a hinge region between globular domains 2 and 3 on CD4 bends to move the HIV envelope trimer closer to CCR5. In turn, a loosening of gp120 allows gp41 to undergo a radical rearrangement that induces the formation of a hydrophobic coiled-coil or six-helix bundle that initiates fusion between viral envelope and cell membrane.

One unanswered question is why the fusion reaction of envelope and cell membrane takes place within endocytotic vesicles (at least in HeLa cell derivatives), since it seems that it is not dependent on low pH [28]. Since X4 viruses readily induce cell-cell fusion, it may well be that these viruses fuse virus and host membranes at the cell surface, whereas R5 viruses may be restricted to undergo fusion in endosomes. A recent paper [29] shows that cortical actin is involved in the pre-fusion conformational changes downstream of gp120-induced signaling via CD4, which promotes HIV entry; abnormally high - or low - levels of gelsolin (which severs cortical actin) inhibit HIV infection.

Much of the spread of HIV infection within an infected person takes place through close contact between cells whereby infected cells form a ‘virological synapse’ with target immune cells [30]. The synapse is held together by adhesion molecules, as well as requiring CD4 and CCR5, and HIV particles move across it from one cell to another. Infected cells can thus spread infection by migrating and disseminating the virus through synaptic contact within the lymph nodes and gut lymphoid tissue where HIV is most likely to encounter activated T cells, in which it replicates best [31].

## Targeting HIV entry in treatment and prevention

Early steps in HIV infection, before the virus has entered cells, are amenable to drugs that are aimed at preventing entry and need not penetrate into the cytoplasm or nucleus [32]. The first potential drug was a soluble form of the CD4 molecule itself that potently neutralized X4 strains, but was only weakly active against R5 strains. However, replacing the head of the heavy chain of IgG with the two amino-terminal domains of CD4 yielded a bivalent protein with more potent anti-HIV activity against both types of virus.

Enfurtide is a 20 amino acid peptide that mimics the fusigenic formation of gp41 sequence and blocks formation of the six-helix bundle (Figure 2), but it requires injection. Perhaps the most promising entry inhibitor approved for clinical use is Maraviroc, which binds to the transmembrane domains of CCR5 and prevents it from acting as an HIV co-receptor.

In theory, drugs such as Maraviroc that are targeted to cellular receptors should not evoke genetic resistance in the virus. However, HIV is artful when under strong selective pressure, and resistance does occur [32]. Mutations in the V3 loop domain of gp120 allow Maraviroc-resistant viruses to interact more strongly with the amino-terminal extracellular domain of CCR5 and become less dependent on the second extracellular loop, which is closer to the drug’s binding site within the transmembrane regions [33].

Our knowledge of HIV receptors and how the envelope glycoproteins interact with them is also relevant for vaccine development [11]. Immunogens that elicit antibodies that block receptor interaction should be protective. Rare monoclonal antibodies derived from naturally infected humans and from experimentally immunized animals recognize the CD4-binding site on gp120. As all the diverse HIV strains utilize CD4, some of these antibodies have breadth as well as potency in neutralizing almost all virus strains. However, designing a vaccine to elicit specifically such antibodies, which occur only rarely and late in natural infections, has proved challenging [34,35].

## Conclusions and prospects

This brief survey has touched upon the very first steps of HIV infection to illustrate how the pathogenesis, development of some types of anti-retroviral drugs and approaches to vaccine discovery have been aided by knowledge of receptors and entry processes. Once HIV gets into the cell, and begins to uncoat and to undergo reverse transcription, interactions with intracellular proteins, including restriction factors, kick in [36], which offer further opportunities for control of HIV infection. As with many viruses, the interaction of HIV with host cell components also illuminates fundamental aspects of cell and molecular biology since viruses are spanners in the works that tell us about the working of the cell.

Looking ahead, HIV entry processes may offer means of prevention of HIV infection in addition to vaccines. For example, blocking receptor interaction could be exploited for the development of novel types of vaginal microbicide based on mini-CD4 molecules and on broadly neutralizing mini-antibodies, which also have potential for blocking mucosal infection [11] of women. The advantage of such anti-HIV neutralizing agents in preventing infection would be to reduce the rapid spread of resistance that will inevitably follow the prophylactic use of the same drugs as are used for treating existing infection.

Yet there remains a huge gap between inventing clever means of blocking HIV infection in the laboratory and translating them into successful public health measures. This is exemplified in HIV vaccine development where the latest clinical trial has just been halted [37] because more cases of HIV-1 infection occurred in the vaccinated arm than in the placebo arm. For an HIV-infected person, being a ‘long-term non-progressor’, with well maintained CD4 cell counts and low viral load, provides an excellent prospect for survival with a reasonable quality of life. But as a vaccine researcher myself, I feel that while we have made interesting scientific discoveries along the way, regrettably we are long-term non-progressors!

## Note

This article is part of the cross journal collection *HIV 30 years on*. Other articles in this series can be found at [38].
